# Developing a comorbidity score in cancer patients using healthcare utilization databases during the COVID‐19 pandemic: An experience from Italy

**DOI:** 10.1002/cam4.5540

**Published:** 2022-12-20

**Authors:** Paolo Lasalvia, Annalisa Trama, Laura Botta, Matteo Franchi, Alice Bernasconi

**Affiliations:** ^1^ Evaluative Epidemiology Unit, Department of Epidemiology and Data Science Fondazione IRCCS Istituto Nazionale dei Tumori Milan Italy; ^2^ National Centre for Healthcare Research and Pharmacoepidemiology University of Milano‐Bicocca Milan Italy; ^3^ Unit of Biostatistics, Epidemiology and Public Health, Department of Statistics and Quantitative Methods University of Milano‐Bicocca Milan Italy

**Keywords:** cancer diagnoses, clinical cancer research, comorbidity score, COVID‐19, epidemiology, healthcare utilization databases, population‐based data, Search Terms: cancer risk factors, statistical methods, risk assessment, risk model

## Abstract

**Background:**

A strong relationship has been observed between comorbidities and the risk of severe/fatal COVID‐19 manifestations, but no score is available to evaluate their association in cancer patients. To make up for this lacuna, we aimed to develop a comorbidity score for cancer patients, based on the Lombardy Region healthcare databases.

**Methods:**

We used hospital discharge records to identify patients with a new diagnosis of solid cancer between February and December 2019; 61 comorbidities were retrieved within 2 years before cancer diagnosis. This cohort was split into training and validation sets. In the training set, we used a LASSO‐logistic model to identify comorbidities associated with the risk of developing a severe/fatal form of COVID‐19 during the first pandemic wave (March–May 2020). We used a logistic model to estimate comorbidity score weights and then we divided the score into five classes (<=−1, 0, 1, 2–4, >=5). In the validation set, we assessed score performance by areas under the receiver operating characteristic curve (AUC) and calibration plots. We repeated the process on second pandemic wave (October–December 2020) data.

**Results:**

We identified 55,425 patients with an incident solid cancer. We selected 21 comorbidities as independent predictors. The first four score classes showed similar probability of experiencing the outcome (0.2% to 0.5%), while the last showed a probability equal to 5.8%. The score performed well in both the first and second pandemic waves: AUC 0.85 and 0.82, respectively. Our results were robust for major cancer sites too (i.e., colorectal, lung, female breast, and prostate).

**Conclusions:**

We developed a high performance comorbidity score for cancer patients and COVID‐19. Being based on administrative databases, this score will be useful for adjusting for comorbidity confounding in epidemiological studies on COVID‐19 and cancer impact.

## Graphical abstract

Developement and validation of a COVID‐19 comordity score for cancer patients. The total cancer patient cohort was split into training (70%) and validation (30%) sets. In the training set, comorbidities associated with the risk of developing a severe/fatal form of COVID‐19 were identified and the score weights were estimated. The score performances were then assesed in the validation set, by areas under the receiver operating characteristic curve (AUC) and calibration plots.

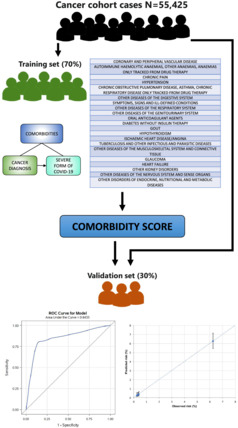

## 1 INTRODUCTION

With the aging of the population, the impact of comorbidities on patients' prognosis is becoming increasingly relevant especially for diseases such as cancer,^1^ for which comorbidities also influence the choice of treatment. Comorbidity scores (e.g., Charlson Comorbidity Index,^2^ Elixhauser Comorbidity Index,^3^ and Chronic‐Related Score^4^) have been developed to stratify the population based on health needs and to adjust for comorbidity confounding in epidemiological studies. However, most of them are not specific to cancer patients, with the exception of the National Cancer Institute (NCI) Comorbidity Index^5^ and the Cancer Multimorbidity Score (CMS).^6^ The NCI Comorbidity Index, however, is based on USA claims^7^ which differ from the administrative datasets available in Europe and Italy. The CMS is a prognostic score focused on elderly patients (aged over 65 years).

In the context of the COVID pandemic, Corrao et al.^8^ developed the COVID‐19 Vulnerability Score, a population‐based risk‐stratification tool capable of predicting severe/fatal clinical manifestations of SARS‐CoV‐2 infection in the general population. Cancer patients are currently known to be more susceptible to SARS‐CoV2 and have a worse prognosis when infected than do the general population.^9^, ^10^, ^11^

Our aim was to develop and validate a comorbidity score to evaluate the impact of comorbidities in developing a severe/fatal form of COVID‐19 in cancer patients (i.e., hospitalization or death from COVID‐19), which will be useful for adjusting for comorbidity confounding in future epidemiological studies on the impact of COVID‐19 and cancer.

## 2 METHODS

### 2.1 Setting and data sources

The study was based on the National Healthcare System beneficiaries of Lombardy Region (approx. 10 million inhabitants), the epicenter of the Italian COVID‐19 pandemic. We used the healthcare utilization databases of Lombardy Region and the COVID‐19 database set up by Lombardy Region during the COVID‐19 pandemic.^12^, ^13^ The COVID‐19 database contains information on infected patients' demographics, swab test results, date of onset of COVID‐19 symptoms, hospital admission, intensive care unit admission, and death due to COVID‐19. The diagnosis of COVID‐19 was swab‐defined or confirmed by the Regional Health Authority. The Lombardy Regional Health Authority interconnected the COVID‐19 database with the healthcare utilization databases through a single identification code. We requested Lombardy Regional Health Authority to provide all healthcare utilization databases from 2009 to the end of 2020 to correctly distinguish incident from prevalent cancer cases. The COVID‐19 database was only available from 2020. We used the latter to retrieve information on COVID‐19 cancer cases who were hospitalized or died due to COVID‐19. We used the healthcare utilization databases to identify incident cancer cases and predictors of severe/fatal COVID‐19, as described in the following paragraphs.

### 2.2 Cancer cohort definition

Using hospital discharge records, we identified a cohort of patients with a diagnosis of a solid cancer (diagnostic codes 140.*‐199.*, according to the International Classification of Diseases, Ninth Revision, Clinical Modification [ICD‐9‐CM]^14^) between February and December 2019, aged over 18 years, as previously described.^15^ In brief, we defined the first admission for a cancer diagnosis as the “index hospitalization”. To select only incident cases, we excluded patients admitted for the same cancer diagnosis as the index hospitalization up to 10 years prior to the index admission. We excluded hematologic neoplasms (ICD‐9‐CM diagnostic codes: 200*‐208*) because these cancers cannot be properly identified by the available databases.

### 2.3 Predictors of severe/fatal COVID‐19

We started from the list of 61 comorbidities initially defined by Corrao et al. We retrieved the comorbidities by searching within 2 years before cancer diagnosis, exploiting hospital discharge records and the drug prescription database.^8^ The hospital discharge records (day hospital and inpatients) contain information on primary diagnosis, coexisting conditions, and provided procedures (coded according to ICD‐9‐CM codes); the drug prescription database provides information on all drugs reimbursed by the National Health System (coded according to the ATC classification system).

### 2.4 Score development

We randomly split the entire cancer patient cohort into two separate datasets: a training set and a validation set. The training set included 70% of the cancer patient cohort followed up to censorship (due to migration or death by other causes), outcome occurrence (i.e., severe/fatal COVID‐19), or to 31st May 2020 (i.e., the end of the first pandemic wave in Italy), whichever happened first. In the training set, we tested each of the 61 comorbidities to exclude multicollinearity by calculating the variance inflation factor (VIF) in a multivariable logistic regression model.^16^ We excluded comorbidities for which <30 patients developed an outcome. We applied the least absolute shrinkage and selection operator (LASSO) method^17^ to select comorbidities independently associated with a severe/fatal form of COVID‐19 up to May 31, 2020 (first wave of the pandemic). The LASSO method shrinks to zero the coefficients of the variables not related to the outcome. We then fitted a multivariable logistic regression model to study the association between age, sex, predictors selected by the LASSO method, and a severe/fatal form of COVID‐19. We assigned a score to each selected comorbidity, multiplying the coefficient estimated by the logistic model by 10 and rounding to the nearest integer. We then added the scores of each comorbidity together to produce the total score. To verify the extension of the association between the increasing values of the score and the increasing occurrence of a severe/fatal form of COVID‐19, we divided the score into classes, according to the quintiles of the predicted probability distribution, obtained by the multivariable logistic regression model.

### 2.5 Score validation and performance

We applied the score developed from the training set to two validation sets. One validation set included 30% of cancer cohort cases with severe/fatal COVID‐19 from March 1, 2020 to May 31, 2020. The other validation set consisted of the cancer cohort cases free from COVID‐19 up to September 30, 2020, followed up from October 1, 2020 to censorship (due to migration or death by other causes), outcome occurrence, or to December 31, 2020, whichever happened first.

We assessed score performance through discrimination and calibration. Discrimination was evaluated by the receiver operating characteristic (ROC) curves and the corresponding underlying areas (areas under the ROC curves (AUCs)).^18^ To confirm the reproducibility of the score even in specific cancer site analyses, we calculated the AUC stratified by the most common cancers (colorectal, lung, female breast, and prostate cancer). We evaluated calibration, that is the ability of the model to assign the correct risk, using calibration plots to visualize observed versus predicted outcome probabilities (reported together with their confidence intervals) for each score class. If the points fall on the x = y line, the model is well calibrated.

We performed all analyses using SAS®Studio and R Statistical Software.

## 3 RESULTS

### 3.1 Cancer cohort cases

We identified 55,425 patients (mean age was 70 years, 53% were males) with a new diagnosis of solid cancer (5762 colorectal, 5457 lung, 8686 female breast, and 5138 prostate cancer cases) between February and December 2019 and a total of 772 severe/fatal COVID‐19 events up to May 31, 2020.

### 3.2 Comorbidity score

We selected 21 out of 61 comorbidities by the LASSO method as independent predictors of severe/fatal COVID‐19 in the training set (Table 1). Cancer patients had three comorbidities on average (data not shown). As expected, respiratory disease, coronary and peripheral vascular disease, hypertension, and chronic obstructive pulmonary disease were the most relevant contributors to the outcome events/severe COVID‐19 (Table 1).

**TABLE 1:** List of comorbidities contributing to the score in the training set (*n* = 38,818 cancer cohort cases), with their absolute (*N*) and relative (%) prevalence, number of outcome events (E), that is severe/fatal COVID‐19 cases, regression coefficient (β), and standard error (SE) estimated by the logistic model and corresponding weights (W) assigned in the score

	*N*	%	E	*β*	SE	W
Coronary and peripheral vascular disease	22,157	57%	340	0.12	0.05	1
Autoimmune hemolytic anemias, Other anemias, Anemias tracked only from drug therapy	13,981	36%	280	0.10	0.05	1
Chronic pain	13,447	35%	214	−0.13	0.05	−1
Hypertension	12,731	33%	231	0.12	0.05	1
Chronic obstructive pulmonary disease, Asthma, Chronic respiratory disease tracked only from drug therapy	7958	21%	140	−0.15	0.05	−2
Other diseases of the digestive system	6690	17%	104	−0.16	0.06	−2
Symptoms, signs, and ill‐defined conditions	6104	16%	109	−0.22	0.06	−2
Other diseases of the respiratory system	6046	16%	418	1.55	0.06	16
Other diseases of the genitourinary system	5332	14%	113	0.09	0.06	1
Oral anticoagulant agents	4529	12%	113	0.16	0.06	2
Diabetes without insulin therapy	4189	11%	83	0.09	0.06	1
Gout	4186	11%	103	0.07	0.06	1
Hypothyroidism	3855	10%	44	−0.14	0.08	−1
Ischemic heart disease/Angina	3101	8%	86	0.11	0.07	1
Tuberculosis and Other infectious and parasitic diseases	3007	8%	105	0.14	0.06	1
Other diseases of the musculoskeletal system and connective tissue	2031	5%	47	0.09	0.08	1
Glaucoma	1932	5%	43	0.16	0.09	2
Heart failure	1752	5%	52	−0.26	0.08	−3
Other kidney disorders	1332	3%	50	0.14	0.08	1
Other diseases of the nervous system and sense organs	1127	3%	45	0.29	0.09	3
Other disorders of endocrine, nutritional, and metabolic diseases	1048	3%	32	0.15	0.10	2

We divided the score into five classes (<=−1, 0, 1, 2–4, >=5). In the training set, 17% of patients had score values ≤−1; 19% had score values = 0; 20% had scores values = 1; 25% had score values between 2 and 4; 19% had score values ≥5. The first four score classes showed similar probability of experiencing the outcome, ranging from 0.2% to 0.5%, while the last score class showed a higher probability, equal to 5.8%.

### 3.3 Score performance

**FIGURE 1:**
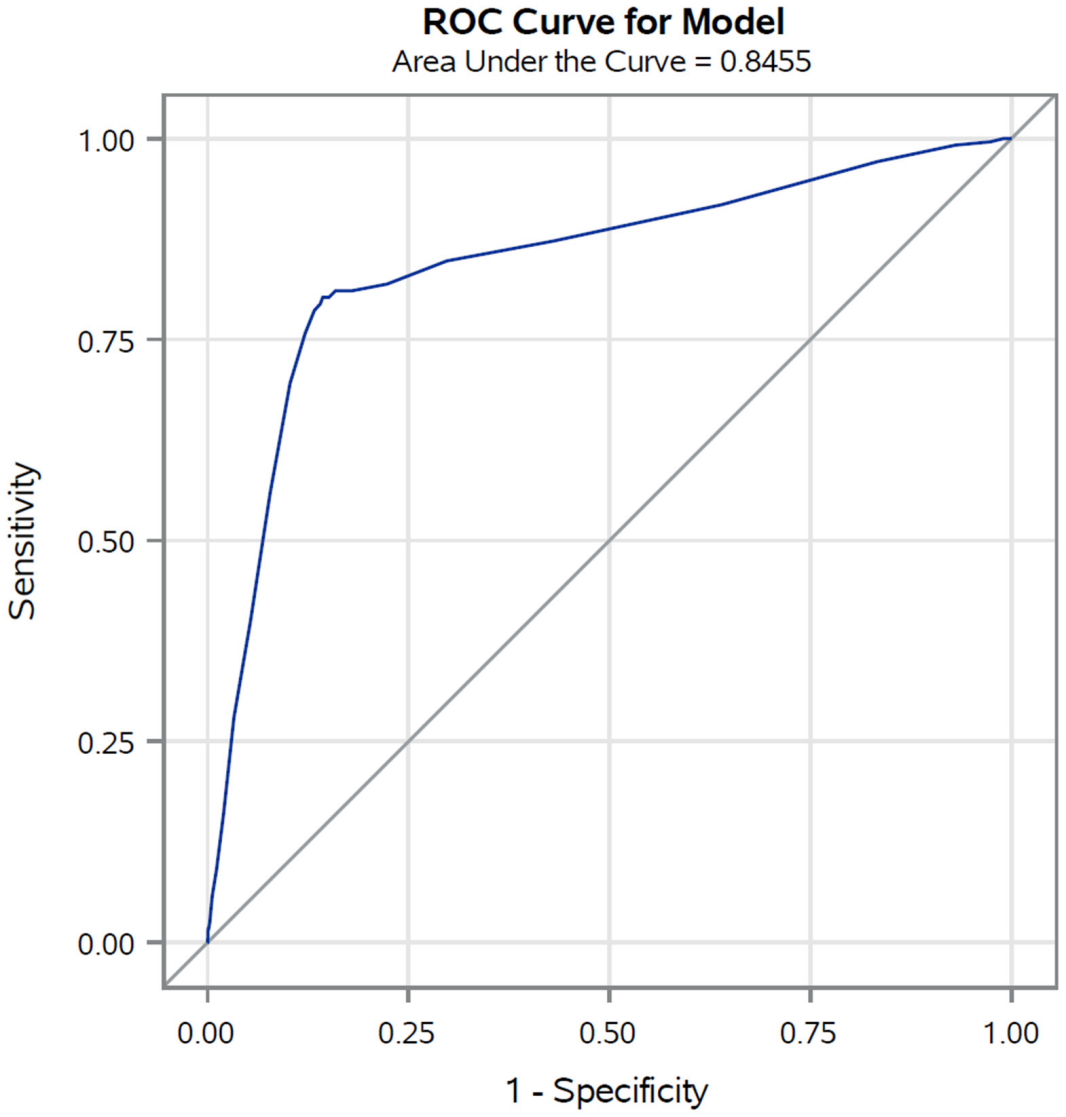
Receiver operating characteristic (ROC) curves and Area Under the Curve (AUC) of the score during the first pandemic wave (March–May 2020)

**FIGURE 2:**
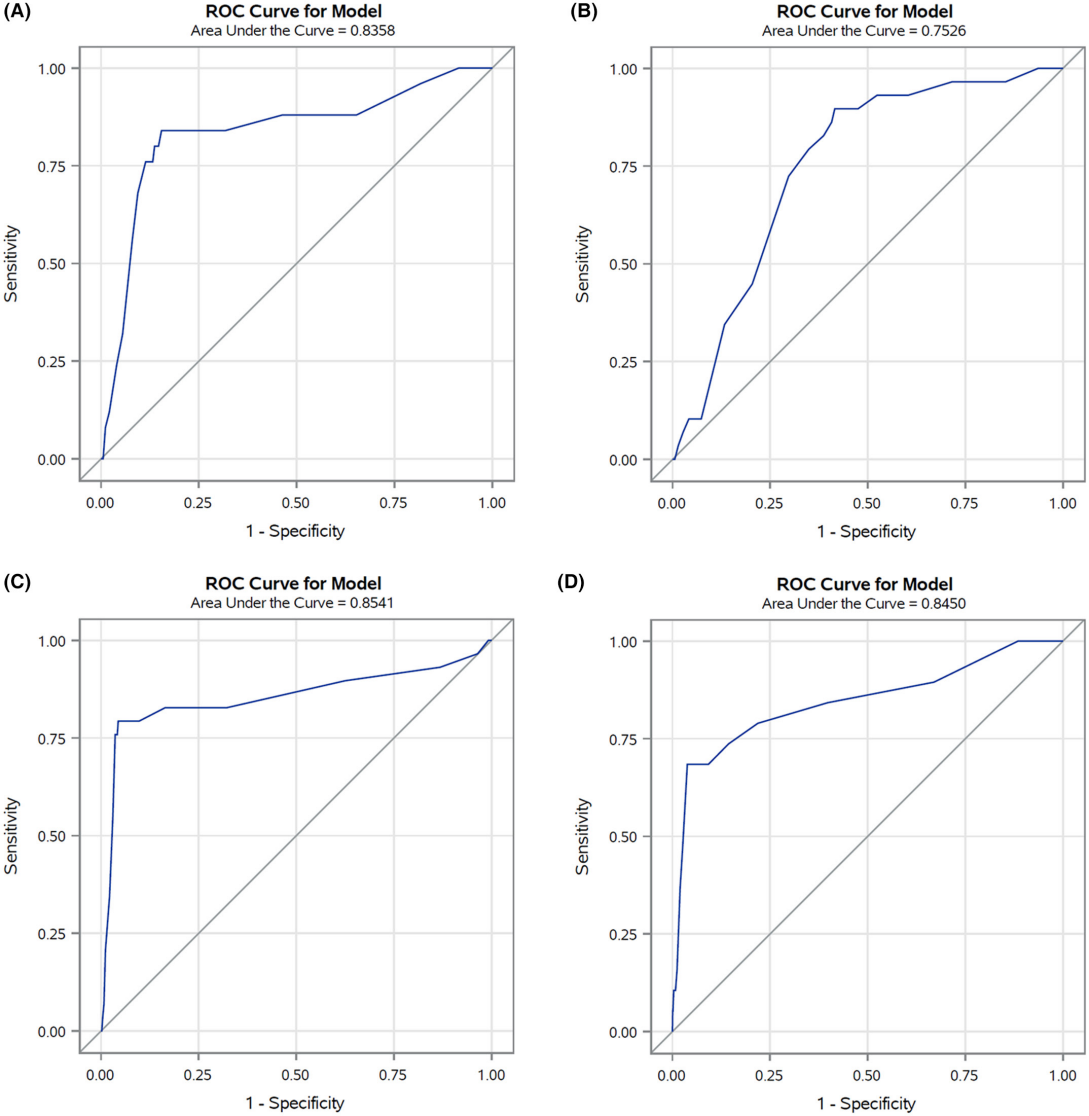
Receiver Operating Characteristic (ROC) curves and Area Under the Curves (AUCs) comparing discriminant power of the score by most common cancers: colorectal (A), lung (B), female breast (C), and prostate cancer (D)

**FIGURE 3:**
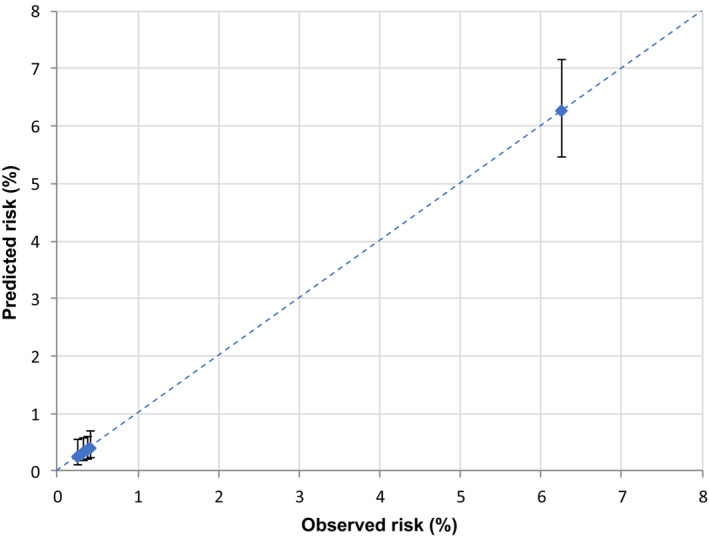
Calibration plot of observed (X‐axis) versus predicted (Y‐axis) percentage (%) risk of a severe/fatal form of COVID‐19, during the first pandemic wave (March–May 2020)

**FIGURE 4:**
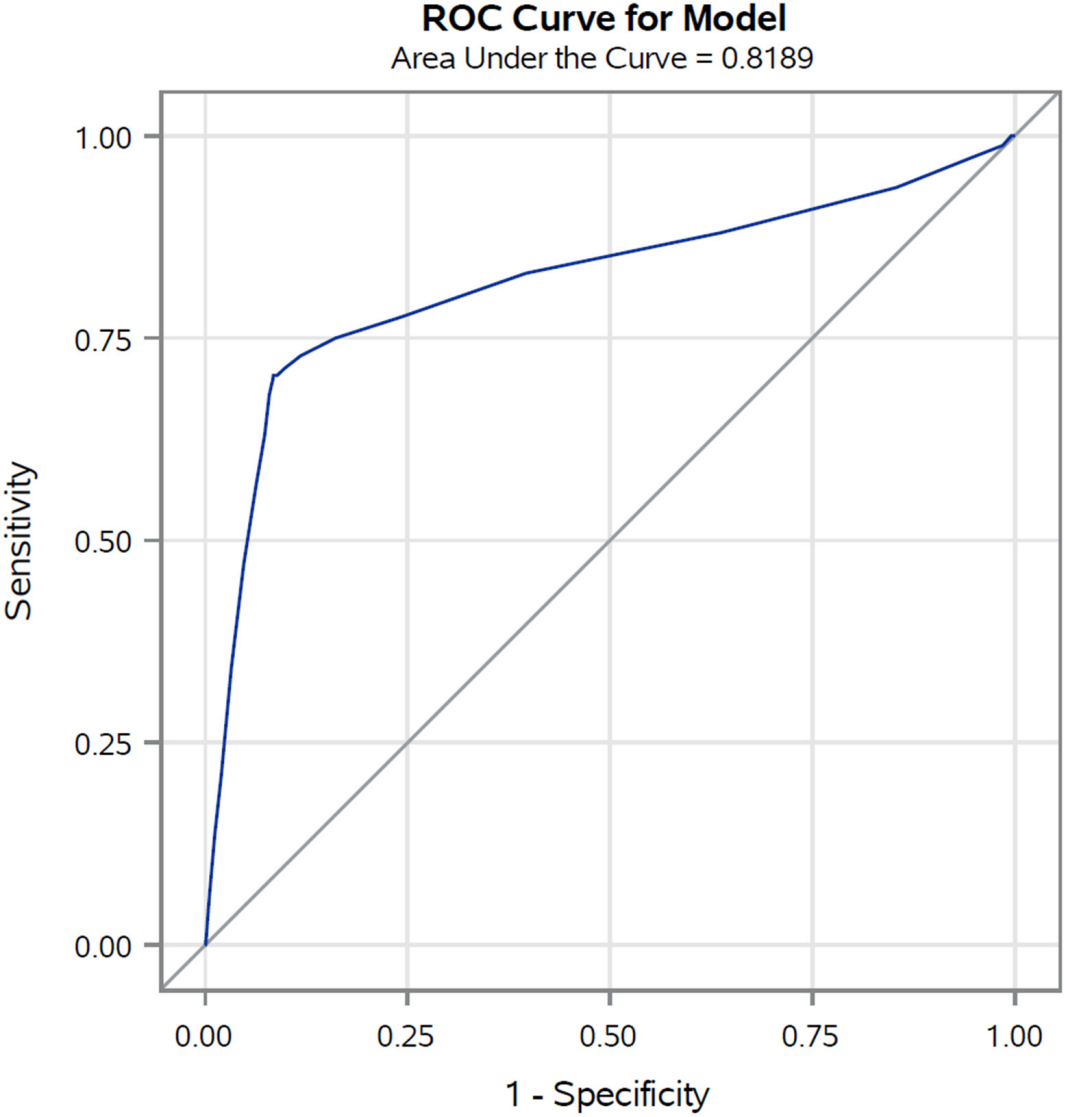
Receiver operating characteristic (ROC) curves and Area Under the Curve (AUC) of the score during the second pandemic wave (October–December 2020)

**FIGURE 5:**
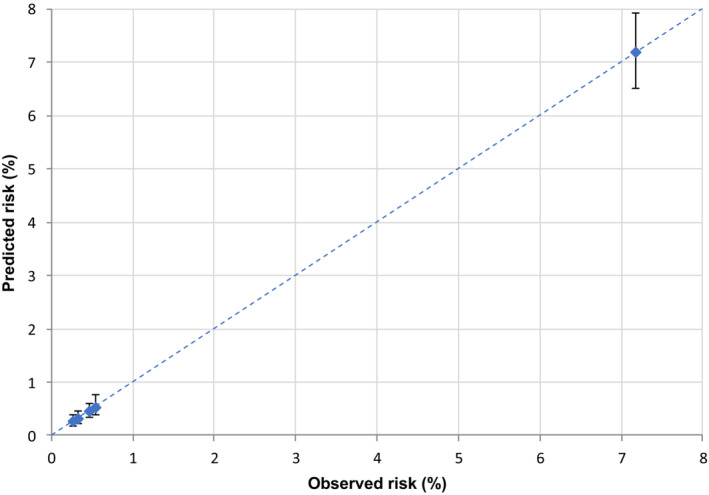
Calibration plot of observed (X‐axis) versus predicted (Y‐axis) percentage (%) risk of a severe/fatal form of COVID‐19, during the second pandemic wave (October–December 2020)

In the validation set (corresponding to 30% of cancer cohort patients with COVID‐19 up to May 31, 2020) the AUC was 0.85 (Figure 1), reflecting high discriminating power. Similar results were also observed for the four primary cancer sites (colorectal 0.84, lung 0.75, female breast 0.85, and prostate 0.84) (Figure 2). The calibration plot (Figure 3) showed good agreement between observed and predicted outcome probabilities, all the points being on the x = y line. During the second wave (October–December 2020) the AUC was equal to 0.82 (Figure 4). The calibration plot (Figure 5) on data of the second wave showed a well calibrated model.

## 4 DISCUSSION

We developed the first score for cancer patients with severe/fatal COVID‐19. Our results showed that the score had high discriminating power in both the first and second wave validation datasets. Moreover, the good AUCs (AUC > 0.75) in the four most common cancers (colorectal, lung, female breast, and prostate cancer) confirmed the adaptability of the score even in specific cancer site analyses. The lowest AUC (0.75), observed for patients with lung cancer, is probably due to the high impact of this poor prognosis tumor on patients' clinical status in addition to existing comorbidities.

Most of the 21 comorbidities included in our score have already been associated with severe form of COVID‐19. According to a recent meta‐analysis,^19^ diabetes, cardiovascular diseases (mainly coronary and peripheral vascular disease), hypertension, chronic respiratory, and kidney diseases were all associated with severe COVID‐19 outcome. People with an infectious disease, such as tuberculosis,^20^ also showed a significant association with the severe form of COVID‐19. Furthermore, other studies have shown that diseases of the neurological system,^21^ metabolism (e.g., gout),^22^ and blood and blood‐forming organs (e.g., anemias)^23^ contributed to clinical frailty related to COVID‐19.

However, the regression coefficients of the 21 comorbidities selected in the logistic model showed different levels of variability, as we can see from the standard error in Table 1. This could be explained by different factors including a few outcomes for a specific comorbidity (e.g., endocrine disorders, nutritional, and metabolic diseases) or autocorrelation of comorbidities within the same patients or across patients, especially in cancer patients with many comorbidities (i.e., the average number of comorbidities in our cohort was three).

Furthermore, during the first wave of the pandemic (i.e., forming our training set), COVID‐19 test availability was limited and prioritized to cases with severe symptoms leading to a possible selection bias for comorbidities and outcome misclassification. However, we also validated our score on the second wave data which confirmed its high discriminating power, albeit slightly lower (AUC ranged from 0.85 to 0.82), and the good ability of our score to assign the true risk to patients. Thus, in both validation sets the equation of the linear model, as graphically illustrated in the calibration plots, included an intercept close to zero and a gradient close to one (dotted line in Figure 3 and Figure 5).

Our score will contribute to assess the impact of COVID‐19 in cancer patients in the short and, more importantly, long term. Thus, by adjusting for several comorbidities related to severe COVID‐19, we will be able to properly disentangle the impact of COVID‐19 on cancer patients' prognosis in future studies.

The present study has several strengths. First, while most of the comorbidity scores were developed from hospital cohorts, making them difficult to adapt to different contexts, our score was based on a large, unselected population. It was also built on healthcare utilization databases widely available in all Italian regions and in several EU Member States. Second, Italian healthcare utilization databases allow services provided by the NHS to be very accurately tracked because suppliers must document their services in order to request reimbursement. Finally, we tested the score in different validation sets and, despite being constructed from Lombardy data collected during the first wave of the pandemic, the score behaved similarly during the second wave, irrespective of differences in treatment options for inpatients and outpatients and in hospitalization criteria compared to the first wave of the pandemic.

The study also has some limitations. We searched for the predictors of severe/fatal COVID‐19 in healthcare utilization databases, thus excluding other factors such as, education, socioeconomic status, functional status, etc. The score also did not distinguish among severity levels of associated comorbidities. We cannot therefore rule out some degree of misdiagnosis resulting from inaccurate reporting of diagnoses and procedures due not only to the purpose of the databases used but also to the emergency situation.

In conclusion, ours is the first score for cancer patients with COVID‐19 that can be used to adjust for confounding comorbidities in future epidemiological studies on the impact of COVID‐19 and cancer. We also confirmed the score's adaptability in the four most common cancers (colorectal, lung, female breast, and prostate cancer). This work emphasizes the importance of promoting data linking and reuse, especially in emergency situations.

## AUTHOR CONTRIBUTIONS

**Paolo Lasalvia:** Formal analysis (lead); investigation (equal); methodology (lead); software (lead); writing – original draft (lead); writing – review and editing (lead). **Annalisa Trama:** Conceptualization (equal); methodology (equal); project administration (lead); writing – original draft (equal); writing – review and editing (equal). **Laura Botta:** Formal analysis (supporting); investigation (supporting); methodology (supporting); software (supporting); writing – original draft (supporting); writing – review and editing (supporting). **Matteo Franchi:** Formal analysis (supporting); investigation (equal); methodology (equal); software (supporting); writing – original draft (equal); writing – review and editing (equal). **Alice Bernasconi:** Formal analysis (equal); investigation (equal); methodology (equal); software (equal); writing – original draft (equal); writing – review and editing (equal).

## FUNDING INFORMATION

This work was supported by “5 per 1000” 2016 funds (Italian Ministry of Health, financial support for healthcare research) – project title “CAncRo E covid (CARE)”, Principal Investigator, Annalisa Trama. The research was also funded by an Italian Ministry of Health “Ricerca Corrente” funds.

## CONFLICT OF INTEREST

All the authors have declared no competing interests.

## ETHICS STATEMENT

The project was approved by the Institutional Review Board and Ethics Committee of the Fondazione IRCCS Istituto Nazionale dei Tumori di Milano, protocol number 0257/20.

## Data Availability

Please contact Regione Lombardia (website: https://www.en.regione.lombardia.it/wps/portal/site/en‐regione‐lombardia) to gain access to the data. Further information is available from the corresponding author upon reasonable request.
